# Different roles for non-receptor tyrosine kinases in arachidonate release induced by zymosan and *Staphylococcus aureus *in macrophages

**DOI:** 10.1186/1476-9255-3-8

**Published:** 2006-05-04

**Authors:** Sandra Olsson, Roger Sundler

**Affiliations:** 1 Department of Experimental Medical Science Lund University, BMC, B12, SE-22184 Lund, Sweden

## Abstract

**Background:**

Yeast and bacteria elicit arachidonate release in macrophages, leading to the formation of leukotrienes and prostaglandins, important mediators of inflammation. Receptors recognising various microbes have been identified, but the signalling pathways are not entirely understood. Cytosolic phospholipase A_2 _is a major down-stream target and this enzyme is regulated by both phosphorylation and an increase in intracellular Ca^2+^. Potential signal components are MAP kinases, phosphatidylinositol 3-kinase and phospholipase Cγ2. The latter can undergo tyrosine phosphorylation, and Src family kinases might carry out this phosphorylation. Btk, a Tec family kinase, could also be important. Our aim was to further elucidate the role of Src family kinases and Btk.

**Methods:**

Arachidonate release from murine peritoneal macrophages was measured by prior radiolabeling. Furthermore, immunoprecipitation and Western blotting were used to monitor changes in activity/phosphorylation of intermediate signal components. To determine the role of Src family kinases two different inhibitors with broad specificity (PP2 and the Src kinase inhibitor 1, SKI-1) were used as well as the Btk inhibitor LFM-A13.

**Results:**

Arachidonate release initiated by either *Staphylococcus aureus *or yeast-derived zymosan beads was shown to depend on members of the Src kinase family as well as Btk. Src kinases were found to act upstream of Btk, phosphatidylinositol 3-kinase, phospholipase Cγ2 and the MAP kinases ERK and p38, thereby affecting all branches of the signalling investigated. In contrast, Btk was not involved in the activation of the MAP-kinases. Since the cytosolic phospholipase A_2 _in macrophages is regulated by both phosphorylation (*via *ERK and p38) and an increase in intracellular Ca^2+^, we propose that members of the Src kinase family are involved in both types of regulation, while the role of Btk may be restricted to the latter type.

**Conclusion:**

Arachidonate release induced by either *Staphylococcus aureus *or zymosan was found to depend on Src family kinases as well as Btk. While members of the Src kinase family were shown to act upstream of Btk and the MAP kinases, Btk plays another role independent of MAP kinases, but down-stream of the Src family kinases.

## Background

Leukotrienes and prostaglandins are important mediators of inflammation, and arachidonate is their precursor. In resident peritoneal mouse macrophages, cytosolic phospholipase A_2 _(cPLA_2_) is the major enzyme responsible for release of arachidonate and this enzyme is regulated by both phosphorylation and an increase in intracellular Ca^2+ ^[1,2].

Zymosan, a cell wall preparation from *Saccharomyces cerevisiae *enriched in mannans and glucans, as well as many bacterial species, are known to elicit arachidonate release in macrophages. There are now several Toll-like and other receptors known that are potentially engaged in initiating this cellular response, but the signalling pathway is not understood in its entirety. We have earlier shown that phosphatidylinositol 3-kinase (PI3K) has an important role in zymosan- and bacteria-induced signalling leading to cPLA_2 _activation by acting upstream of phospholipase Cγ2 (PLCγ2) [3], which becomes activated via tyrosine phosphorylation and/or translocation to the membrane after stimulation with zymosan.

The products of the PLC reaction result in activation of protein kinase C, with the subsequent activation of the MEK/ERK pathway and an increase in cytosolic Ca^2+^, respectively. Both of these events will lead to activation of cPLA_2_. The MAP kinases ERK and p38 both contribute to the activation of cPLA_2 _in response to zymosan or the Gram-negative bacterium *Prevotella intermedia *[4] and the downstream kinase MAP kinase signal-integrating kinase-1 (Mnk-1) has been proposed to play a role in the phosphorylation of cPLA_2 _[5].

The PLC family includes three subgroups (β, γ and δ) and PLCγ is known to undergo tyrosine phosphorylation, possibly as part of its activation. The tyrosine kinase(s) involved in PLCγ activation are not clearly identified, but the Src family kinases (SFK) are candidates since PLCγ is a possible substrate [6]. Members of the SFK are known to play a critical role in many signaling pathways, with a putative role in inflammation. Furthermore, SFK have been shown to interact with both PLCγ [7,8] and PI3K [9-11]. However, it is not known whether SFK are involved in responses induced in macrophages by zymosan or bacteria. As a key downstream target for SFK, Btk, a member of the Tec kinase family, may be important in receptor dependent signalling in a variety of hematopoietic cell lineages [12], but if it plays a role in the eicosanoid response in macrophages is unknown. The role of Btk has been underlined by phenotypic analysis of cells with naturally occurring mutations in Btk, such as those from the *xid *mouse [13,14]. These studies show that Btk is important downstream of the B-cell antigen receptor, where PI3K and SFK function upstream of Btk [15]. To determine the role of SFK two different inhibitors with broad specificity (PP2 and the Src kinase inhibitor 1, SKI-1) were used. This is of particular importance due to the frequent occurrence of redundancy among the individual SFK [16,17].

Our results indicate that SFK and Btk play differential roles in arachidonate release induced by *Staphylococcus aureus *(*S. aureus*) and yeast-derived zymosan beads and that SFK act upstream of PI3K, PLCγ2 and MAP kinases, whereas Btk plays a separate role independent of MAP kinases, but down-stream of the SFK.

## Methods

### Materials

Zymosan, SU6656 and wortmannin, were from Sigma Chemical Co. (St Louis, MO, USA). Heat-killed *S. aureus *was kindly provided by Dr Lars Björck, BMC, Lund University. PP2 was from Biomol (Plymouth Meeting, PA, USA), while SP600125 was from Tocris Cockson (Northpoint, UK). LFM-A13 and Src kinase inhibitor I (SKI-1) from Calbiochem (La Jolla, CA, USA). [^3^H]Arachidonic acid (196 Ci/mmol) was from Amersham international (Little Chalfont, UK). Antibodies against p-JNK, PLC-γ2, ERK-2 and phosphotyrosine as well as HRP conjugated secondary antibodies, were from Santa Cruz Biotechnology (Santa Cruz, CA, USA). Antibodies against p-ERK, p-p38 and pAkt (Thr308) were from New England Biolabs (Beverly, MA, USA). Antibodies against pAkt (Ser473) and pBtk (Tyr223) were from Cell Signaling Technology (Beverly, MA, USA). The Mnk-1 inhibitor CGP057380 was a kind gift from Dr Hermann Gram, Novartis Pharma AG, Basel, Switzerland. Zymosan and bacteria were dispersed in PBS. Zymosan was used at 0,2 mg/ml and bacteria at a concentration of 2,5*10^7/ml. The inhibitors LFM-A13, PP2, CGP057380, SU6656, SKI-1 and wortmannin were dissolved in dimethyl sulphoxide (DMSO) and added to cells resulting in a final concentration of DMSO of less than 0.3%.

### Isolation and culture of macrophages

Peritoneal macrophages were isolated from female outbred NMRI mice by adherence in a humidified atmosphere of 5% CO_2 _in air at 37°C. Non-adherent cells were removed after 2 h and the rest of the cells were incubated in medium 199 supplemented with 1% fetal bovine serum for 18–20 h. During this incubation cells were labelled with [^3^H]arachidonic acid in some experiments.

### Release of [^3^H]arachidonate

Cells labelled with 0,5 μCi [^3^H]arachidonic acid/well for 18–20 h, were washed three times with PBS, and stimulated in serum-free medium. At the end of the experiment the medium was collected and the cells scraped off the dishes in 0,1% Triton X-100 in water. The collected medium was centrifuged and the arachidonate released from cellular phospholipids was determined by liquid scintillation counting. The release into the collected medium is expressed as a percentage of total recovered radioactivity in cells and medium.

### Immunoblotting

Cells were cultured in 10 cm^2 ^dishes and stimulated in a serum-free medium. When inhibitors were used, they were added 15 min. before stimulus. After stimulation the cells were scraped off the dishes in 110 μl sample buffer and equal amounts of cell lysate were subjected to SDS-PAGE. The proteins were transferred to polyvinylidene fluoride (PVDF) membranes which were blocked in 3% or 5% non-fat milk/gelatin for 1 h followed by incubation with different antibodies. Bound antibodies were detected with secondary horseradish peroxidase-labeled antibodies and enhanced chemiluminescence using LAS 1000 Plus (Fuji Film, Stockholm, Sweden). To assure equal loading of the gels the membranes were stripped and reprobed with ERK-2 antibody, or as indicated.

### Immunoprecipitation

Macrophages (approx. 10^6 ^cells) were stimulated in serum-free medium. When inhibitors were used, they were added 15 min. before the stimulus. At the end of the experiment cells were washed with ice-cold PBS and scraped off the dish in lysis buffer (0.1 M TrisCl pH 7.4, 150 mM NaCl, 1 mM EDTA, 1 % nonionic detergent (Igepal, polyoxyethylene nonylphenol), 20 mM NaF, 1 mM Na_3_Vo4, 1 mM PMSF and 1 μg/ml each of pepstatin and leupeptin). After centrifugation (10^4 ^× g) for 10 min at 4°C the supernatant was incubated with an antibody against PLC-γ2. The immune complexes were captured using protein A-Sepharose (50% w/v). The samples were centrifuged and the immune complexes were washed three times in lysis buffer. The immunoprecipitated proteins were dissolved in 2X sample buffer and subjected to SDS-PAGE (7% acrylamide) and analyzed by Western blotting.

## Results

### Src family kinases transmit signal(s) to arachidonate release by acting upstream of MAP kinases

**Figure 1:**
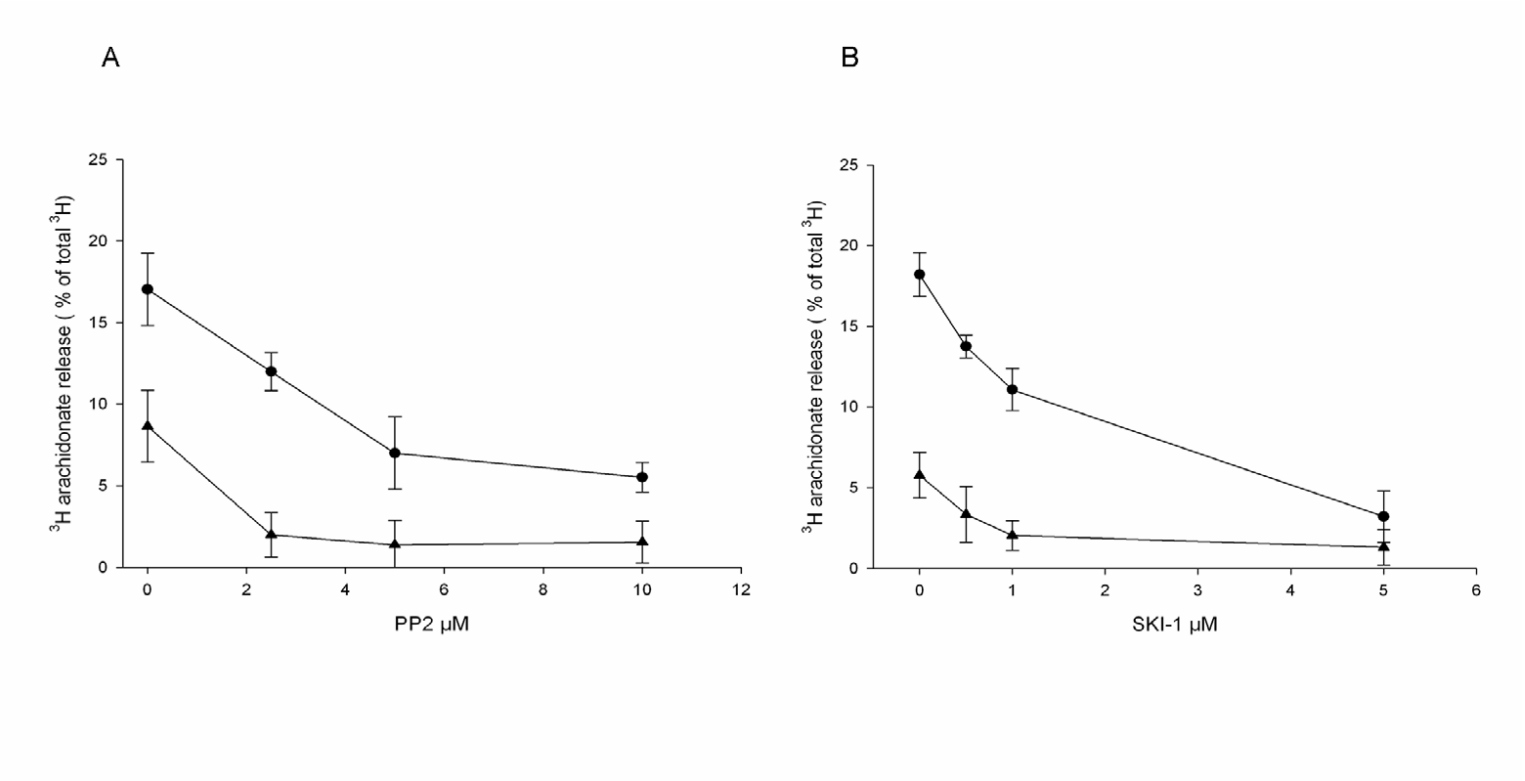
**Inhibition by PP2 or SKI-1 of zymosan- and *S.aureus*-induced release of arachidonate**. Macrophages were labeled with [3H]arachidonic acid for 20 h. The cells were pretreated for 15 min with the indicated concentrations of PP2 **(A) **or SKI-1 **(B) **followed by stimulation with either zymosan (●) for 45 min or *S.aureus *(▲) for 60 min. Results are mean ± SEM (n = 3) and corrected for the release in control cultures.

Both zymosan and Gram-negative bacteria induce arachidonate release in mouse macrophages [4], zymosan being the more potent of the two classes of stimuli. SFK are important in many signalling pathways, but their role in the present process is so far unknown. To investigate this two different Src kinase inhibitors (PP2 and SKI-1) were used. Both SFK inhibitors reduced the response induced by zymosan and Gram-positive *S. aureus *in a concentration dependent manner (Fig. 1). The decrease at 5 μM PP2 was approximately 59% for zymosan and 84% for *S. aureus*. SKI-1 (5 μM) inhibited the zymosan-induced release by approximately 82% and the *S. aureus-*induced release by 83%.

**Figure 2:**
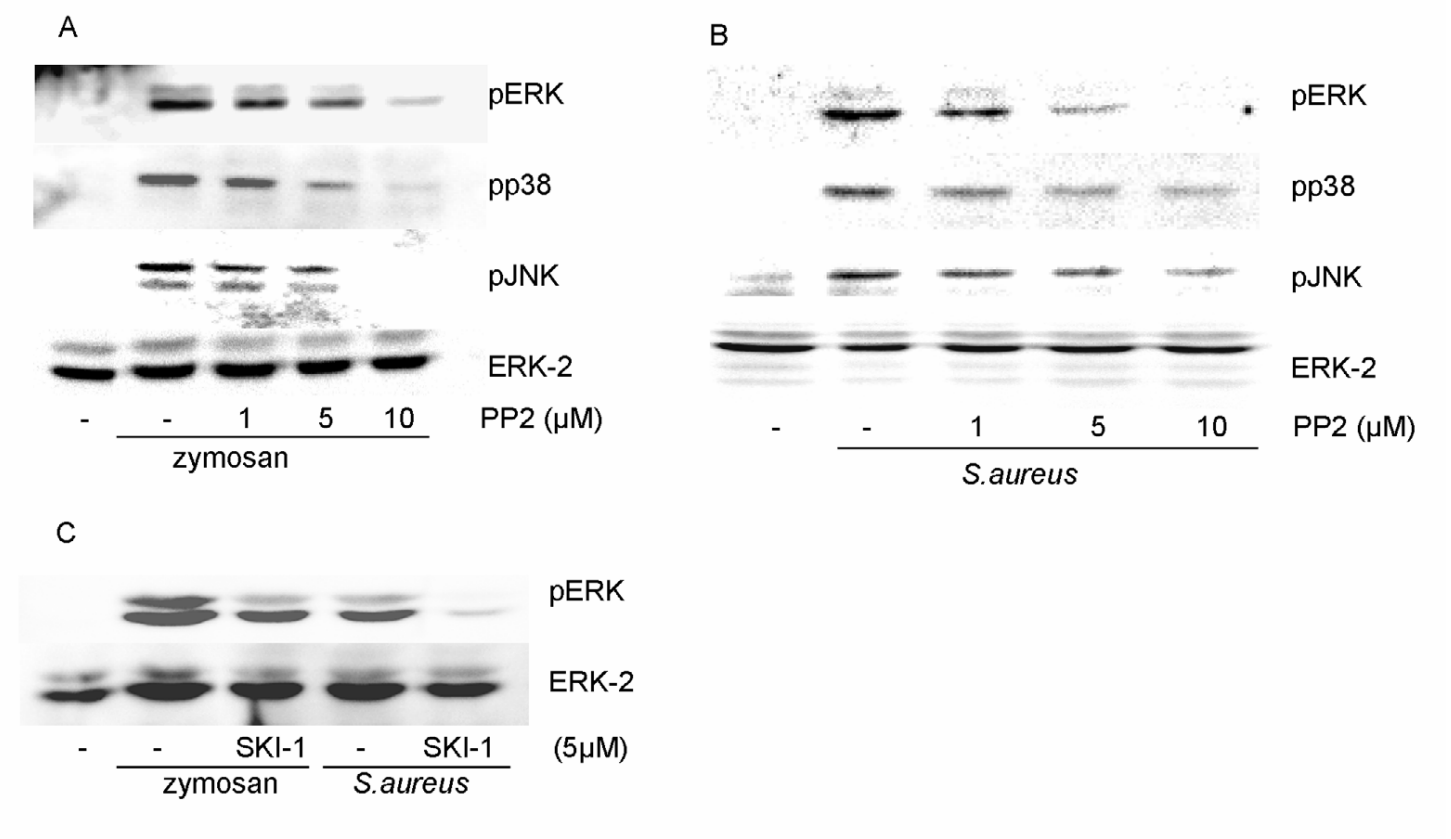
**Inhibition by PP2 or SKI-1 of zymosan- and *S.aureus*- induced phosphorylation of MAP kinases**. Macrophages were pretreated for 15 min with PP2 (1–10 μM), followed by stimulation with zymosan (**A**) or *S.aureus *(**B**) for 20 min. (**C**) Macrophages were pretreated for 15 min with SKI-1 (5 μM), followed by stimulation with zymosan or *S.aureus *for 20 min. Equal amounts of cell lysate were run on 10% polyacrylamide gels and probed with phosphospecific antibodies against ERK, p38 and JNK. The membrane was reprobed with ERK-2 antibody to verify equal loading of protein.

The MAP kinases ERK and p38 are both known to contribute to the activation of cPLA_2 _in response to zymosan or bacteria [4]. Both zymosan and whole bacteria induce regulatory phosphorylations of these MAP kinases and we show here that pretreatment of the cells with PP2 (1–10 μM) decreased these phosphorylations in the case of not only ERK and p38, but also of JNK (Fig. 2A, B). Also SKI-1 was able to decrease the zymosan- and bacteria-induced phosphorylation of ERK (Fig. 2C).

To establish whether also JNK might play a role in the activation of cPLA_2 _SP600125, a JNK inhibitor [18] was tried. Pretreatment with SP600125 (5–40 μM) led to a concentration dependent decrease of the zymosan-induced arachidonate release. However, this was accompanied by a parallel inhibition of the phosphorylation of the MAP kinases ERK, p38 and JNK as detected by antibodies against the phosphorylated MAP kinases (not shown). Thus, under our conditions SP600125 may also act as an inhibitor upstream of several MAP kinases.

It has been suggested that the kinase Mnk-1, downstream of both ERK and p38, might transmit the signal to some of the phosphorylations necessary for the activation of cPLA_2 _[5]. Treatment of cells with the Mnk inhibitor CGP 57380 (20 and 40μM) resulted in partial inhibition (41 and 50%, respectively) of the arachidonate release in response to zymosan (Table 1).

**Table 1:** Effects of the Mnk-1 inhibitor CGP057380 on arachidonate release induced by zymosan. Macrophages were labelled with [3H]arachidonic acid for 20 h. The cells were pretreated for 15 min with the indicated concentrations of CGP507380 followed by stimulation with zymosan for 45 min. The release of radiolabel is expressed as percentage of total cellular ^3^H. The results are expressed as mean ± S.E.M. (n = 3).

[^3^H]Arachidonate release (% of total ^3^H)		
(n = 3)		
		
-	20 μM	40 μM
12.9 ± 0.9	7.6 ± 0.9	6.5 ± 1.4

### Zymosan-induced phosphorylation of Akt and PLCγ2 depend on Src kinases

**Figure 3:**
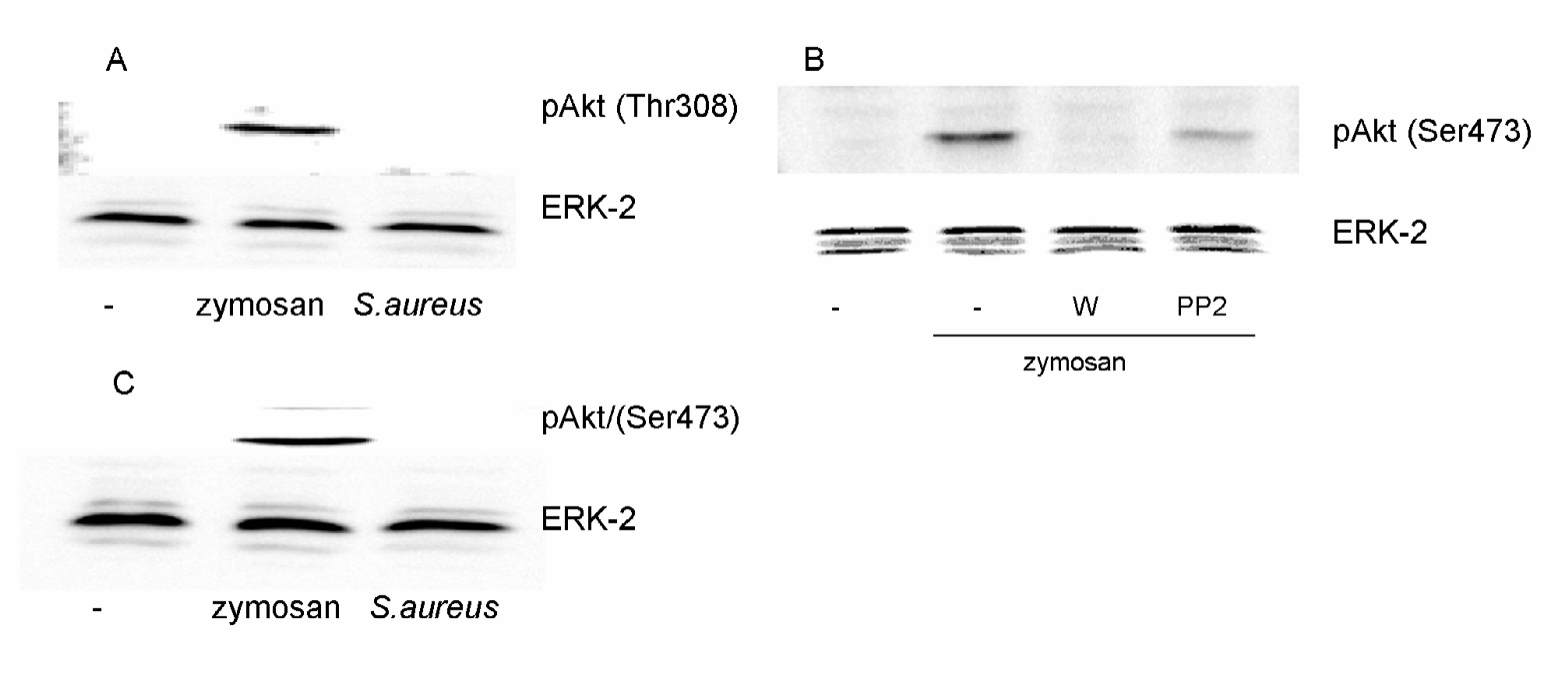
**Effect of inhibitors against PI3K, Src family kinases and Btk on the tyrosine phosphorylation of Akt**. Zymosan but not *S. aureus *induce tyrosine phosphorylation of Akt. This phosphorylation was affected by inhibitors against PI3K and Src family kinases. **(A and C) **Macrophages were stimulated for 30 min with either zymosan or heat killed *S.aureus*. **(B) **Macrophages were pretreated for 15 min with either PP2 (5 μM) or wortmannin (W, 100 nM), followed by stimulation with zymosan for 30 min. Cell lysates were processed for immunoblotting with the indicated antibody as described in Methods. The membrane was reprobed with ERK-2 antibody to verify equal loading of proteins. The data are representative of three separate experiments.

We have previously shown that PI3K has an important role in the zymosan and bacteria induced signalling to cPLA_2 _activation and arachidonate release [19]. PI3K can cause activation of the serine/threonine protein kinase Akt. The activation is dependent on phosphorylation of two sites, one in the activation loop of the kinase core (Thr 308) and one near the carboxy terminus (Ser 473). Zymosan beads, in contrast to *S. aureus*, induced Akt phosphorylation at both Ser 473 and Thr 308 in macrophages, as detected by phosphospecific Akt antibodies (Fig. 3A). The phosphorylation of both sites appeared to depend on SFK activation and PI3K. This can be seen as decreases in these phosphorylations after inhibition with either PP2 or wortmannin (Fig. 3B).

**Figure 4:**
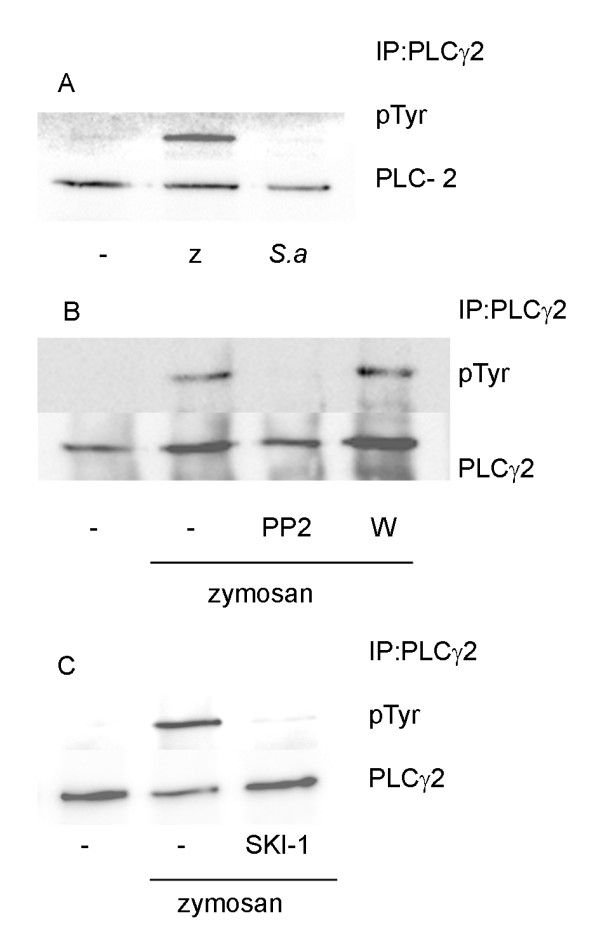
**Zymosan but not *S.aureus *induced tyrosine phosphorylation of PLC γ2**. (**A**) Macrophages were stimulated with zymosan(z) or *S.aureus *(*S.a*) for 45 min. **(B and C) **Macrophages were pretreated for 15 min with either PP2 (5 μM), wortmannin (W, 100 nM) (**B**) or SKI-1 (5 μM) (**C**) followed by stimulation with zymosan for 30 min. Cell lysates were immunoprecipitated with antibody against PLCγ2 as described, followed by Western blot analysis with phosphotyrosine-specific antibody. The membrane was stripped and reprobed with antibody against PLCγ2. The data are representative of three separate experiments.

PLCγ2 is considered to be activated by tyrosine phosphorylation and/or by the product of PI3K, but both PI3K-dependent and -independent pathways leading to activation have been demonstrated. The role of Src kinases in zymosan-induced PLCγ2 activation was investigated by immunoprecipitation with a PLCγ2 specific antibody. A prominent tyrosine phosphorylation was induced by zymosan, but was not detected in cells exposed to *S. aureus *(Fig. 4A). Pretreatment of the cells with PP2 (Fig. 4B) or SKI-1 (Fig. 4C) clearly inhibited the zymosan-induced tyrosine phosphorylation of PLCγ2. In agreement with previous findings [3], inhibition of PI3K with wortmannin did not cause such inhibition (Fig. 4B).

### MAP kinase-independent role of Btk down-stream of Src kinases

**Figure 5:**
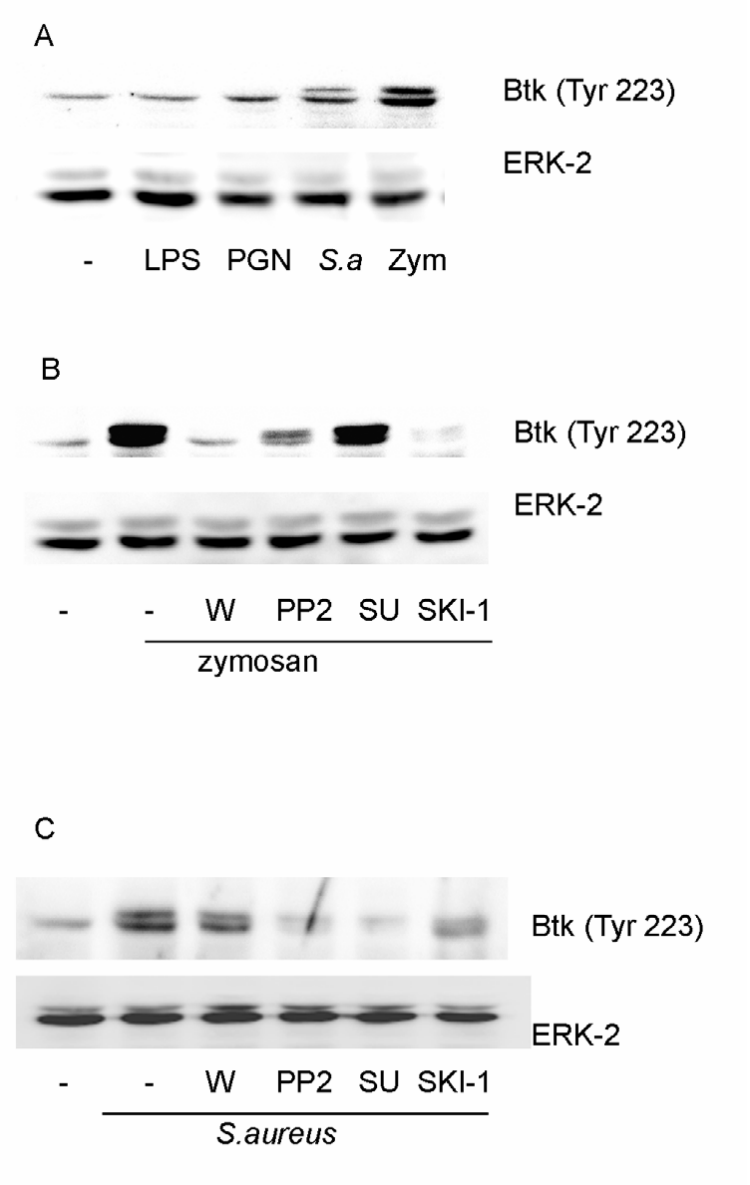
**Zymosan and bacteria but not LPS or peptidoglycan (PGN) induce phosphorylation of Btk**. (**A**) Macrophages were stimulated with zymosan (zym), *S.aureus *(*S.a*.), LPS or PGN for 30 min. **(B and C) **Macrophages were pretreated for 15 min with either wortmannin (W, 100 nM), PP2 (5 μM), SU6656 (5 μM) or SKI-1 (5 μM), followed by stimulation with zymosan (**B**) or *S.aureus *(**C**) for 30 min. Equal amounts of cell lysate were run on polyacrylamide gels and probed with phosphospecific antibodies against Btk. The membrane was reprobed with ERK-2 antibody to verify equal loading of proteins. The data are representative of three separate experiments.

Btk is a member of the Tec family of cytoplasmic tyrosine kinases. Most studies on Btk have been conducted with B-lymphocytes, where cell activation leads to membrane translocation of Btk and phosphorylation of two sites (Tyr 551 and Tyr 223). Tyr 551 is situated in the activation loop and its phosphorylation may be initiated by SFK, leading to autophosphorylation of Tyr 223, which appears necessary for full activation [20,21]. Detection of Btk activation by phosphospecific (Tyr 223) antibody showed that a low level of phosphorylation on Tyr 223 was present in control cells (Fig. 5A). Furthermore, zymosan and *S. aureus *but not LPS or peptidoglycan induced phosphorylation of Btk in macrophages leading to enhanced immunostaining of the band detectable in control cells as well as appearance of an additional band, presumably due to additional phosphorylation(s) causing gel-shift (Fig. 5A). Pretreatment with the PI3K inhibitor wortmannin or the SFK inhibitors PP2 and SKI-1 caused total, or pronounced, inhibition of zymosan-induced phosphorylation of Btk (Fig. 5B). A Src kinase inhibitor with a different specificity (SU6656;[22]) was much less inhibitory (Fig. 5B). A different inhibitor profile was observed when *S. aureus *was used as stimulus (Fig. 5C). Btk phosphorylation (on Tyr223) was then insensitive to wortmannin, but sensitive to all three SFK inhibitors, including SU6656. These results, together with differences in the phosphorylation of Akt (Fig. 3A) and PLCγ2 (Fig. 4A) argue for differential engagement of individual SFK members as well as of PI3K in the response to zymosan and *S. aureus*.

**Figure 6:**
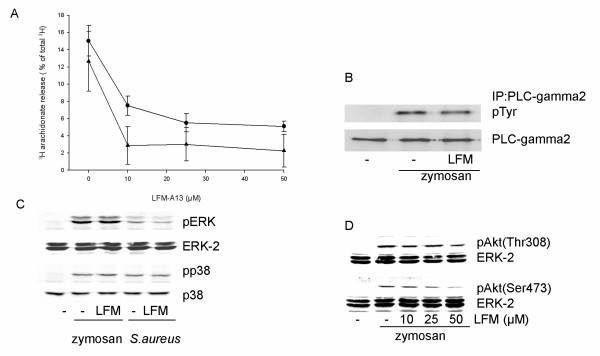
**Effects of Btk inhibitor on arachidonate release and the phosphorylation of PLCγ2, Akt and MAP kinases**. (**A**) Macrophages were labeled with [3H]arachidonic acid for 20 h. The cells were pretreated for 15 min with the indicated concentrations of LFM-A13 followed by stimulation with either zymosan (●) for 45 min or *S.aureus *(▲) for 60 min. Results are mean ± SEM (n = 3) and corrected for the release in control cultures. (**B**) Macrophages were pretreated for 15 min with LFM-A13 (25 μM), followed by stimulation with zymosan for 30 min. Cell lysates were immunoprecipitated with antibody against PLCγ2 followed by Western blot analysis with phosphotyrosine-specific antibody. The membrane was stripped and reprobed with antibody against PLCγ2. (**C**) Macrophages were pretreated for 15 min with LFM-A13 (25 μM), followed by stimulation with zymosan or *S.aureus *for 20 min. Equal amounts of cell lysate were run on 10% polyacrylamide gels and probed with phosphospecific antibodies against ERK and p38. The membrane was reprobed with ERK-2 antibody to verify equal loading of protein. (**D**) Macrophages were pretreated for 15 min with LFM- A13 (25 μM) followed by stimulation with zymosan for 30 min. Western blot analysis was performed with phosphospecific antibodies against Akt. The membrane was reprobed with ERK-2 antibody to verify equal loading of protein. Data shown in B-D are representative of three separate experiments.

Interestingly, an inhibitor of Btk (LFM-A13) caused a decrease in the release of arachidonate induced by both zymosan and *S. aureus *(Fig. 6A). The decrease was prominent at 10 μM LFM-A13 with little further change at higher concentrations (approx. 65% inhibition for zymosan and ≥ 80% for *S .aureus *at 50 μM LFM-A13). However, in contrast to the effect of SFK inhibitors described above, the inhibition of Btk did not affect the zymosan- and bacteria-induced phosphorylation of ERK and p38 (Fig. 6C), neither did it affect the zymosan-induced phosphorylation of PLCγ2 (Fig. 6B). A partial inhibition of the phosphorylation of Akt induced by zymosan was observed (Fig. 6D), but only at higher concentrations than required for inhibition of arachidonate release (Fig. 6A).

These data indicate that Btk has a regulatory role for arachidonate release, is acting downstream of inhibitor-sensitive SFK, but not involved in signalling to MAP kinase activation.

## Discussion

In this report we provide evidence that signalling to release of arachidonate induced in resident mouse peritoneal macrophages by non-opsonized zymosan (yeast cell-wall particles) and the Gram-positive bacterium *S. aureus*, is differentially dependent on SFK and the Tec kinase Btk. Src kinases act upstream of both Btk and the MAP kinases ERK and p38, thereby also of activating phosphorylation(s) of cPLA_2_. They are also most likely responsible for the tyrosine phosphorylation of PLCγ2 that occurs in response to zymosan, as shown here and previously [3]. Btk is important for arachidonate release, but independent of the MAP kinase cascade.

The major enzyme responsible for release of arachidonate in the cells used in the present study is cPLA_2_, which is regulated by both phosphorylation(s) and an increase in intracellular Ca^2+ ^[1,2]. Several sites on cPLA_2_, especially in the C-terminal cluster of serine residues [2], become phosphorylated upon agonist stimulation and the protein kinase Mnk-1 has been suggested to be involved in the phosphorylation of one of these (Ser 727) [5]. Our finding that a direct inhibitor of this kinase reduced zymosan-induced arachidonate release is consistent with the suggestion. Mnk-1 is coordinately regulated by the MAP kinases ERK and p38 and inhibition of both of these MAP kinases severely inhibits zymosan-induced arachidonate release [4]. However, separate inhibition of either of the two kinases argues for a more prominent role for ERK than p38 [4]. The SFK inhibitor PP2 counteracted bacteria- and zymosan-induced phosphorylation of both ERK and p38. PP2 has been shown to inhibit human p38 with similar potency as the SFK member Lck [23] which could, potentially, influence the interpretation of our data on arachidonate release. However, the pronounced inhibition by PP2 of the activation of MAP kinases, including p38, makes any direct inhibitory effect on p38 subordinate. A similar inhibitory effect on MAP kinase phosphorylation/activation was exerted by SKI-1, as illustrated by its effect on ERK. SFK are previously known to regulate MAP kinase activation (see [6] for review). In contrast, inhibition of Btk did not inhibit the MAP kinase cascade.

PI3K has an important role in zymosan- and bacteria-induced signalling in macrophages [19]. SFK apparently affect the zymosan-induced phosphorylation of Akt, a downstream kinase of PI3K, indicating that one or more of these tyrosine kinases are situated upstream of PI3K. SFK and PI3K may interact in several ways; it is known that the p85 subunit of PI3K is able to interact with both the SH3 and SH2 domain of Src[9] and it is known that the p85 subunit can function as a substrate for SFK [6]. Furthermore, the p85 subunit of PI3K is known to interact with phosphotyrosine residues on different adaptor proteins. Binding of PI3K to such residues or a tyrosine kinase at the membrane is likely to help position the catalytic subunit of PI3K to its lipid substrate.

We now demonstrate that the tyrosine phosphorylation of PLCγ2 induced by zymosan is dependent on SFK, as shown by its sensitivity to the inhibitors PP2 and SKI-1. PLCγ is a possible substrate for Src [6] and the activation of PLCγ was blocked by PP1 (another Src kinase inhibitor) both in muscle cells from chicken embryos [7] and in FDC-P1 cells stimulated by EPO [24]. Furthermore, Src activation has been shown to induce calcium release via a PLCγ dependent mechanism in *Xenopus *egg extracts [8]. These results all indicate that SFK are important regulators of PLCγ. Because the phosphorylation of PLCγ2 is insensitive to wortmannin, as shown here as well as previously [3], the effect of PP2 is probably not mediated through PI3K but either direct or mediated by another kinase. It should be emphasized, though, that the role of tyrosine phosphorylation of PLCγ2 in the regulation of its activity remains unclear (see [25] for review). *S.aureus *did not induce detectable tyrosine phosphorylation of PLCγ2. Nevertheless both phosphorylation of ERK and arachidonate release induced by this bacterium were sensitive to wortmannin (data not shown) and therefore most likely mediated via PI3K and accompanied by activation of PLCγ2.

We also found that inhibition of Btk did not affect the zymosan-induced tyrosine phosphorylation of PLCγ2 in macrophages. Most studies on Btk have been carried out in B cells, while information about the role of Btk in macrophage signaling is scarce. Btk activation in B-cells is known to affect both PI3K and Ca^2+ ^levels and Btk activation results in a rise in the level of IP_3 _and depletion of intracellular calcium stores [26]. Furthermore, Btk regulates PtdIns4,5P_2 _synthesis which may affect both Ca^2+^-signaling and PI3K activity [27]. Btk can associate with PtdIns4P-5kinases, enzymes that synthesize PtdIns4,5P_2_, and upon activation generate local PtdIns4,5P_2 _synthesis [27]. PtdIns4,5P_2 _is a substrate not only for PI3K but also for PLCγ2 and increased synthesis may well be necessary to provide substrate for PLCγ2 and also be of importance for optimal generation of PtdIns3,4,5P_3 _[27]. A difference between the phosphorylation of PLCγ2 by zymosan in macrophages and its phosphorylation after B cell receptor cross-linking is the PI3K dependency. Zymosan-induced PLCγ2 phosphorylation is not inhibited by wortmannin [3], whereas PLCγ2 phosphorylation in B cells is PI3K dependent [26,28]. In view of our own data and the findings referred to above, we propose that Btk may primarily affect arachidonate release *via *the generation or further metabolism of PtdIns4,5P_2 _and the cellular Ca^2+^-homeostasis.

## Conclusion

**Figure 7:**
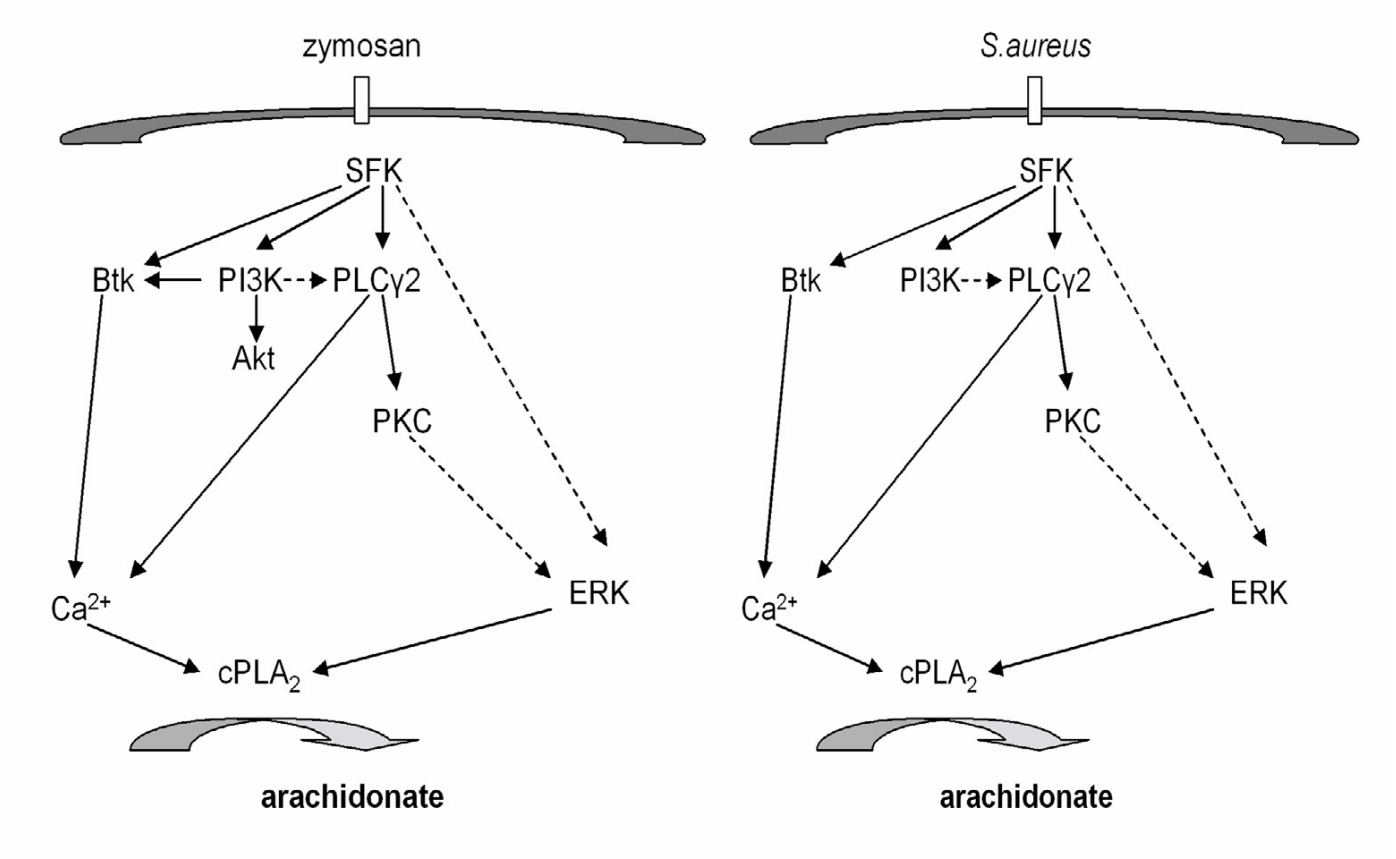
**Summary of the role of Src kinases and Btk**. Schematic illustration of signaling pathways involved in the activation of cPLA_2 _and arachidonate release in resident mouse macrophages responding to zymosan or *S.aureus *and the role of Src family kinases (SFK) and Btk. Broken arrows delineate proposed connections, based on previous or present evidence, that remain to be confirmed.

Arachidonate release initiated in mouse macrophages by either *S. aureus *or yeast-derived zymosan beads was found to depend on SFK members, in part with agonist-specific differences, as well as the Tec kinase Btk. While Src kinases were shown to act upstream of Btk, PI3K, PLCγ2 and the MAP kinases ERK and p38, Btk was not involved in the activation of ERK and p38. An attempt to summarise the findings is provided (Fig. 7).

## Competing interests

The author(s) declare that they have no competing interests.

## Authors' contributions

SO participated in the design and execution of all experiments and helped to draft the manuscript. RS participated in the design of the study and in the preparation of the manuscript. Both authors read and approved the final manuscript.
